# Intestinal Colonization of Preterm Neonates with Carbapenem Resistant Enterobacteria at Hospital Discharge

**DOI:** 10.3390/antibiotics12020284

**Published:** 2023-02-01

**Authors:** Vera Mijac, Snezana Brkic, Marija Milic, Marina Siljic, Valentina Cirkovic, Vladimir Perovic, Milos Markovic, Ivana Cirkovic, Maja Stanojevic

**Affiliations:** 1 University of Belgrade, Faculty of Medicine, Institute of Microbiology and Immunology, Department of Microbiology, 11000 Belgrade, Serbia; 2 Institute for Laboratory Diagnostics Konzilijum, 11000 Belgrade, Serbia; 3 Institute of Neonatology, 11000 Belgrade, Serbia; 4 University of Belgrade, Faculty of Medicine, Institute of Microbiology and Immunology, Department of Immunology, 11000 Belgrade, Serbia

**Keywords:** carbapenem resistant Enterobacterales, carbapenemases, colonization, preterm neonates, duration of carriage

## Abstract

Our aim was to investigate gut colonization with carbapenem-resistant Enterobacterales (CRE) in the population of preterm neonates at discharge from a tertiary care center in Serbia. The study included 350 randomly selected neonates/infants discharged in the period April 2018–May 2019. CRE colonization was present in 88/350 (25.1%) of patients. *Klebsiella pneumoniae* producing KPC and OXA-48 carbapenemase were detected in 45 and 42 subjects, respectively, while NDM producing *Escherichia coli* was identified in one patient only. All OXA-48 strains harbored *bla*_CTX-M-15_, while both *bla*_TEM_ and *bla*_SHV_ were present in all but one KPC-producing strain. CRE isolates exhibited a multidrug resistance pattern with uniform fluoroquinolone resistance, universal susceptibility to colistin, and variable susceptibility to aminoglycosides. Administration of carbapenems was common (~50%) and it was strongly associated with colonization, as well as the combinational therapeutic regimens that included meropenem, contrary to ampicillin–sulbactam/colistin therapy and prolonged course of the initial therapy (ampicillin/amikacin ≥ 7 days). Other risk factors for CRE carriage were level of immaturity, admission to neonatal intensive care unit, prolonged hospitalization and invasive procedures. Although the rate of clinically and/or laboratory proven systemic infections was significantly higher among colonized patients, CRE infection was confirmed in one patient only (1.1%) that was colonized with NDM *E. coli*. Clonal relatedness of CRE isolates was high, with seven and eight clusters detected among KPC (N = 30) and OXA-48 (N = 37) producing strains, respectively. The follow up of the 31 KPC-colonized patients after discharge from hospital revealed common decolonization within one month (~68%). In conclusion, our results demonstrated a high rate of CRE colonization that is most likely related to carbapenem consumption and lack of screening as important infection prevention practice.

## 1. Introduction

Carbapenem-resistant Enterobacterales (CRE) have emerged as a major health threat. Their global dissemination is mainly due to the presence of carbapenemases that are encoded by genes located on mobile genetic elements. The most important carbapenemase types include *Klebsiella pneumoniae* carbapenemase (KPC), New-Delhi metallo-β-lactamase (NDM), Verona integron-encoded metallo-β-lactamase (VIM), imipenemase or IMP-type metallo-β-lactamase (IMP) and oxacillinase-48 (OXA-48) [1]. CRE may colonize the human gut, but also have a potential to cause infections and outbreaks that are difficult to treat, as therapeutic options are rather limited [2]. The risk of colonization and/or subsequent infection is higher among hospitalized patients, especially those with underlying conditions requiring invasive procedures, antibiotic treatment and prolonged hospital stay [3,4]. CRE are increasingly reported in health care settings worldwide, including pediatric and neonatal wards [5,6]. Premature neonates (born before 37 weeks of gestation) comprise a vulnerable population that frequently requires long-term hospitalization due to the overall immaturity, underdeveloped mucosal surfaces and need for invasive manipulations, such as total parenteral nutrition, central and peripheral venous lines, respiratory support and stay in neonatal intensive care units (NICU). Contact with hospital environment and medical personnel, contaminated surfaces and supportive equipment increases exposure to multidrug-resistant (MDR) bacteria, accruing the risk of acquisition and transmission of CRE in this population [3,7]. Infections and outbreaks due to CRE are gradually more reported in preterm neonates [6,8]; however, information regarding CRE colonization in this population is scarce [9]. Few data available have shown that higher incidences of colonization (up to 60%) with CRE are present among neonates in less developed areas [10,11], compared to the industrialized countries where the incidences of CRE colonization among children in general, are rather low [9]. The importance of CRE carriage is also in the risk of transmission to other patients, as it represents a reservoir of resistant bacteria. Transmission may remain unnoticed in settings where screening for colonization is not regularly performed. Analysis of clonal relatedness of isolates from a single setting is important to estimate the proportion of cross transmission and frequency of introduction of new strains into hospital, in order to inform the hospital infection prevention team aimed to control CRE dissemination. Of note, CRE carriage may persist after discharge from hospital and lead to spill-over of resistant bacteria to other individuals and in the community [12]. Knowledge about the duration of CRE colonization is, therefore, of paramount significance to assess the risk of spread; however, information about it is globally very scarce [13], especially in the population of preterm neonates. So far, there are no data regarding CRE colonization in any patient population in Serbia. Our previous study conducted at a tertiary neonatal care center has shown a high level of colonization with MDR bacteria in the first week of hospitalization among preterm neonates; nevertheless, CRE was not detected [14]. Therefore, our aim was to determine the incidence of gastrointestinal tract colonization with CRE among premature neonates/infants at discharge from a tertiary neonatal care center (Institute of Neonatology, Belgrade, Serbia), to explore the bacterial genetic basis of resistance, genetic relatedness of strains and to evaluate risk factors associated with colonization. In addition, the duration of CRE carriage was evaluated on the follow up visits after one, three, six and twelve months. 

## 2. Results

### 2.1. Incidence and Characteristics of Patients Colonized with CRE 

Out of 350 infants screened at the hospital discharge, 88 (25.1%) were colonized with CRE. Patients’ demographic and clinical data are presented in Table 1.

Overall, CRE colonized patients had significantly lower birth weight (<1000 g), lower first minute Apgar score, and were in need of umbilical venous catheter placement more often, compared to the non-colonized ones. A significant majority of CRE colonized patients (75%) were admitted to NICU (*p* < 0.001) and their in-hospital stay was significantly longer than non-colonized patients (*p* < 0.001). Further on, mechanical ventilation, total parenteral nutrition, and lower level of maturity (≤32 weeks of gestation) were more common among the colonized; however, the difference did not reach statistical significance. The number of neonates with laboratory confirmed sepsis was significantly higher in CRE colonized patients while the rate of clinical sepsis without laboratory confirmation did not differ between the groups. Moreover, the complications associated with preterm birth were overall more frequent among colonized neonates, but statistical significance was found for necrotizing enterocolitis (NEC) and intraventricular hemorrhage (IVH) (*p* = 0.001 and *p* = 0.002, respectively).

Although, laboratory confirmed sepsis was significantly more common among CRE colonized (*p* < 0.001), infection due to CRE was confirmed in only one colonized patient (1.1%) with symptoms of pneumonia, whose tracheal aspirate taken at hospital admission yielded *Escherichia coli* with resistance patterns identical as the colonizing strain detected at hospital discharge.

### 2.2. Antibiotic Therapy in Regard to Colonization with CRE

Initial antibiotic therapy, as a regular procedure at admission was administered to all patients during the first 5–7 days of hospitalization, whereas in one third of patients initial therapy was given for longer period (7–15 days) (Table 2). Additional (second line) antimicrobial therapy was administered to 56% of patients.

Carbapenems (meropenem) were the most frequently used, either as monotherapy or in combination with other agents, and they were applied to almost half of all patients in the study (49.4%). In a univariate logistic model, carbapenem administration was significantly associated with CRE colonization, as well as all combination therapies that included meropenem (Table 2). 

Identification of CRE strains showed that among 88 isolates there were 87 *Klebsiella pneumoniae*, and one *E. coli*. All CRE isolates carried carbapenemase genes with predominance of OXA-48 and KPC-2 carbapenemases that were detected in 45 (51.1%) and 42 (47.7%) strains of *K. pneumoniae*, respectively, while NDM-1 was found in a single *E. coli* strain (Table 3). Extended spectrum beta lactamase (ESBL) genes were also present in all CRE isolates, but their distribution differed between OXA-48 and KPC isolates. All OXA-48 producing strains harbored *bla*_CTX-M-15_ type ESBL, while all KPC strains harbored both *bla*_TEM_ and *bla*_SHV_ ESBLs with the exception of one strain that lacked SHV enzyme. Antimicrobial susceptibility and resistance patterns of CRE isolates are presented in Table 3.

### 2.3. Clonal Relatedness of KPC and OXA-48 Carbapenemase Producing Isolates

**Figure 1:**
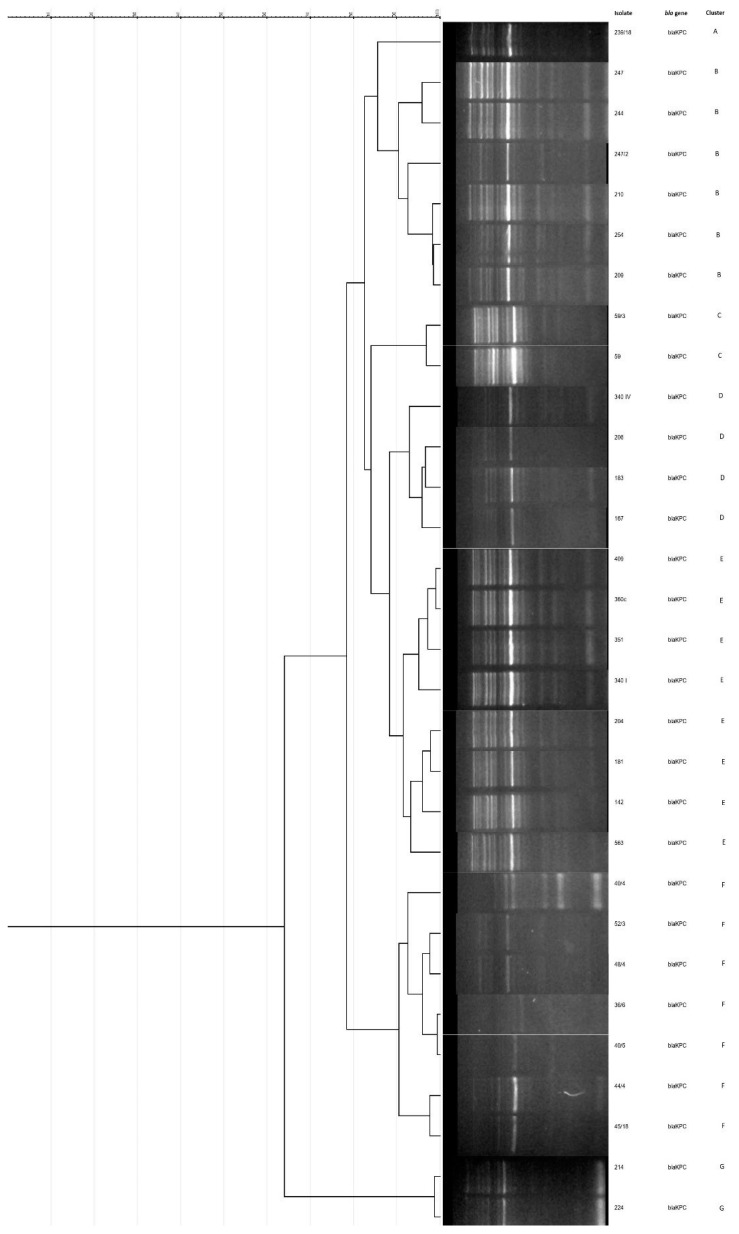
Dendrogram and gel image obtained by ERIC-PCR analysis of *bla*_KPC_-positive *Klebsiella pneumoniae* isolates (N = 30). Seven different clusters (A–G) were generated at the similarity level of 90%. ERIC-PCR, Enterobacterial Repetitive Intergenic Consensus PCR.

**Figure 2:**
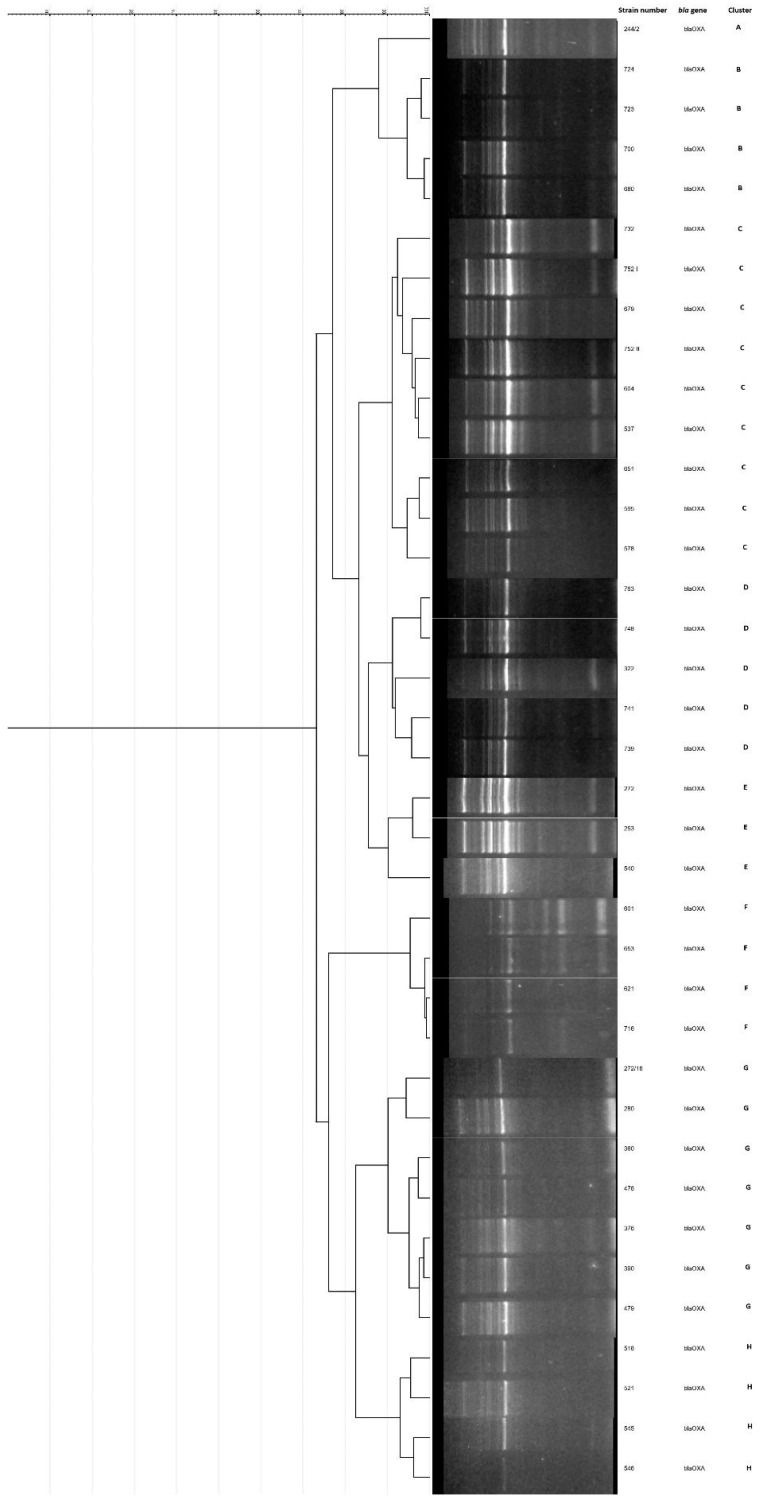
Dendrogram and gel image obtained by ERIC-PCR analysis of *bla*_OXA-48_-positive (N = 37) *Klebsiella pneumoniae* isolates. Eight different clusters (A–H) were generated at the similarity level of 90%. ERIC-PCR, Enterobacterial Repetitive Intergenic Consensus PCR.

Phylogenetic analysis of *bla*_KPC_-positive isolates of *K. pneumoniae* (N = 30) have showed seven clusters (A–G). Six clusters were comprised of two or more (maximum eight) isolates, while one represented a singleton (Figure 1). Among *bla*_OXA-48_ positive isolates (N = 37), eight clusters (A–H) were determined, with three to nine isolates, and with one singleton (Figure 2).

### 2.4. Duration of Colonization with KPC Carbapenemase Producing Isolates

**Figure 3:**
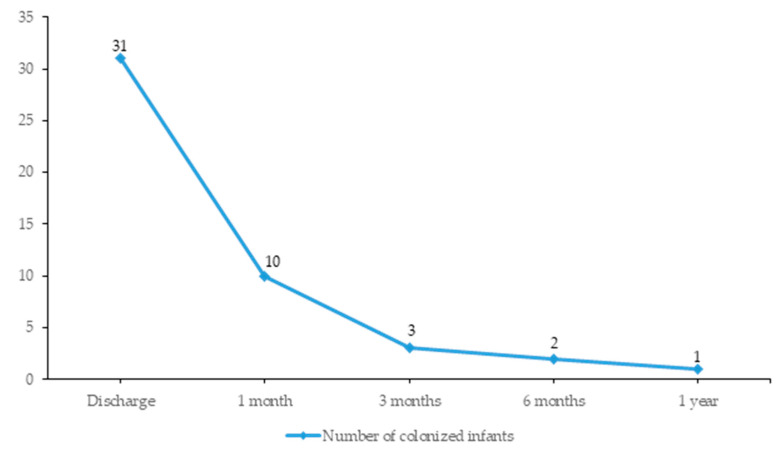
Time to negative culture of KPC-producing *Klebsiella pneumoniae* during follow-up visits.

Out of 42 patients colonized with KPC producing *K. pneumoniae* at discharge from the Institute of Neonatology, 31 came to the routine follow-up visits (at one, three, six and twelve months after discharge) when carriage of CRE was screened. The majority of neonates/infants (21/31, 67.8%) were culture negative one month after discharge, and positivity rate diminished slightly at every subsequent control visit, while 1/31 patients (3.2%) remained colonized one year post-discharge (Figure 3).

## 3. Discussion

The present study provided the first data on the prevalence, pattern and risk factors associated with CRE colonization among preterm neonates treated at a tertiary care center in Belgrade, Serbia, with the obtained results implicating an alarmingly high burden of CRE colonization in the study population. Worldwide, existing data on CRE carriage in preterm neonates indicate high occurrence in low income countries, reaching as much as 65% in some countries in eastern and southeastern Asia, and the gradual increase in the incidence during hospitalization [3,10,11,15]. On the other hand, in the USA, during a five year period, the rate of CRE colonization among hospitalized children was 4.5% [9], and low incidence were also found in Western European countries [16]. Risk of dissemination of CRE and other resistant bacteria among preterm neonates is elevated compared to other populations [5,17]. Predisposing factors include general immaturity of mucosal barriers and other organ systems, weakened immunity and exposure to a hospital environment which fundamentally influences microbiota formation [17]. Carriage is especially more probable in settings lacking screening [18]. All the aforementioned aspects may also have contributed to our findings. Many risk factors for CRE colonization were identified in this study with important ones being lower birth weight and Apgar score, NICU admission, prolonged hospitalization, invasive manipulations (umbilical venous catheter, UVC placement) and NEC. Longer NICU stay, mechanical ventilation and total parenteral nutrition were also more frequent among CRE carriers; however, the difference did not reach statistical significance. The prominent finding in the present study was very high antibiotic usage in our patients, that indicates the role of antibiotic exposure as a major factor in selection and propagation of resistant strains. Nearly half of the patients in our study received carbapenem therapy, which was found to be significantly associated with CRE colonization. Furthermore, colonized patients were more likely to receive any of the frequently applied antibiotic regimens that include meropenem, compared to the non-colonized patients. On the other hand, prolonged initial therapy (ampicillin plus amikacin), although frequently administered (to one third of patients), as well as treatment comprising ampicillin–sulbactam/colistin combination, were not identified as a risk factor for CRE colonization in our study. Our findings are in line with other studies showing that antibiotic therapy, and carbapenem-containing regiments in particular, pose a significant predisposing factor for the acquisition of CRE [7,13,15]. 

CRE carriage, especially in susceptible populations, is considered to be a risk factor for subsequent infection [4,17,18]. Even though laboratory confirmed blood infections (sepsis) were more frequent among colonized patients in our study, none of them was due to CRE. There was only one case of CRE infection in a neonate diagnosed with pneumonia at admission to the hospital. Culture of a patient’s tracheal aspirate showed presence of carbapenem-resistant *E. coli* that was also detected in the patient’s rectal swab at discharge. The observed discrepancy between colonization rate and confirmed infection could be attributed to the varying virulence of particular CRE strains, with the possibility that not all strains may have propensity to cause disease. It is also probable that carbapenem therapy administered for suspected/confirmed infections facilitated propagation of CRE and resulted in high rate of CRE carriage. Moreover, if CRE have reached endemic level in this institution, transmission might have been enhanced by the selective pressure of antibiotic regiments, as well as other factors.

As for carbapenemase distribution, CRE have already been reported in Serbia [19], with previous data pointing towards predominance of OXA-48 and NDM and sporadic appearance of KPC among clinical isolates, while, up until now, no data on CRE colonization in any target population existed in the country. In this study, a predominance of OXA-48 and KPC producing *K. pneumoniae* was identified. Major ESBL genes were detected in all CRE and their distribution was uniform (*bla*_CTX-M-15_ among all OXA-48 strains and *bla*_TEM_ and *bla*_SHV_ in majority of KPC-producing isolates). 

Clonal relatedness of carbapenemase-producing *K. pneumoniae* was high, suggesting intrahospital origin and cross transmission in hospital wards. Among *bla*_OXA-48_-positive isolates, 56.7% were grouped in three clusters, while isolates with *bla*_KPC_ showed more distinct clonality, with 70% of the isolates distributed in three clusters and singletons were detected in both KPC and OXA-48 strains. Tendency of clonal spread of *K. pneumoniae* in health-care institutions has already been well described [5,17] even in the settings with intensified infection control measures [20], with possibility of acquisition of new strains. A single NDM *E. coli* strain identified in our study was most probably introduced to the NICU from obstetric health-care institution since it was detected in tracheal aspirate on patients’ admission, with no evidence of further spread. Antimicrobial susceptibility patterns of our CRE isolates showed multidrug resistance (MDR), including fluoroquinolone resistance, even though the latter agents are not given to neonates. Gentamicin/amikacin and chloramphenicol were active in most of the cases in spite of exposure of all patients to amikacin or gentamicin during the course of the initial therapy, while susceptibility to colistin was universal. According to the surveillance data obtained through the Central Asian and European Surveillance of Antimicrobial Resistance (CAESAR) network, the burdens of CRE and antimicrobial resistance in Serbia are generally high [21], which is consistent with our results. 

Regarding duration of carriage, in our study, in the majority of patients, clearance of colonization with KPC-producing CRE was achieved within one month upon discharge from hospital. However, KPC *K. pneumoniae* positivity remained detectable in the study group throughout the study period, including one patient (~3%) who remained colonized until the last control visit, after a year of follow-up. While information about the length of CRE carriage in infants is largely lacking, data from the adult population show that spontaneous decolonization occurs after prolonged periods of time from 3 months [22] up to a year [13], with repeated hospitalizations, infections due to CRE and carbapenem usage increasing the risk of persistent colonization [13]. Possible cause and long term implications of the specific colonization dynamics in the study population still remain to be explored. Of note, lack of hospitalization history in other health-care settings between discharge and follow-up visits, and the relatively small number of infants included in the follow-up may be considered a limitation of the presented study. However, a tendency towards shorter persistence of CRE in premature infants compared to adults, may be explained by the distinctive characteristics of this population. Since in prematurely born children the initial encounter with microorganisms and acquisition of microbiota begin in the hospital environment, it is plausible to assume that gut microbial composition might easily change after exposure to the household environment. However, more studies are warranted to explore in detail the dynamics of CRE colonization in infants.

In conclusion, this is the first study in Serbia and one of the very few studies in general exploring the occurrence and persistence of CRE colonization in the population of preterm neonates/infants. Our data indicate that the rate of CRE colonization among preterm infants on hospital discharge from a tertiary care center in Serbia is linked to high exposure to carbapenems, together with NICU admission, prolonged hospitalization and overall immaturity of premature neonates. On the other hand, low incidence of laboratory confirmed CRE infection, as well as short persistence after discharge imply possible lower pathogenic potential of colonizing CRE strains and highlights the need for further studies aimed to clarify the relation between colonization and infection due to CRE. Our results also indicate that practice of antibiotic stewardship and implementation of screening as a preventive measure is of utmost importance in the control of the spread of MDR pathogens in neonatal wards and avoidance of possible selection of highly resistant pathogens in the future. 

## 4. Materials and Methods

The Institute of Neonatology is the only tertiary neonatal care center at the country level aimed at providing long-term neonatal/infant health care to premature neonates born in obstetric wards all over the country, with number of admissions in the range of 700–900 per year. The standard procedure for all patients on admission includes sampling of blood culture and administration of initial antibiotic therapy (ampicillin plus amikacin or gentamicin, further referred to as initial therapy), applied until a negative result of blood culture is obtained (approximately for up to 5 days). Placement of UVC is performed on admission for low weight neonates (<1600 g) and for all patients in the NICU. For disinfection, alcohol or alcohol-based combinations are used on skin, for personnel hand hygiene, and daily medical devices and surfaces disinfection, while quaternary ammonium compounds in combination with formic acid and/or alcohol are used for floors. So far, monitoring of colonization with resistant bacteria is not implemented on admission or at any point during hospitalization.

Out of 869 patients discharged between April 2018 and May 2019, 350 premature born neonates/infants were randomly selected and included in the study. On discharge from the hospital, rectal swabs were taken, placed into Amies transport medium (Copan, Italy), and transported to a microbiology laboratory. From CRE colonized patients that came to the routine follow-up visits (intended after one, three, six months and twelve months), rectal swabs were taken as described, to determine the duration of colonization. Data on patients’ demographics, as well as clinical data after delivery, on admission to the Institute of Neonatology and during hospitalization (antimicrobial therapy, surgical procedures, presence of indwelling devices, respiratory support, microbiology results, etc.) were obtained from medical records. The signed parental/caregiver informed consent to participate in the study was obtained for each patient and the study protocol was approved by the institutional Ethics Committees.

Screening for CRE intestinal colonization was performed by inoculating rectal swabs on CHROMID CARBA and CHROMID OXA-48 media (BioMerieux, Lion, France). After overnight incubation, suspected colonies were identified by conventional biochemical tests and API 20E (BioMerieux, France). Antimicrobial susceptibility testing and confirmation of a carbapenem-resistant phenotype was performed according to the European Standard for Antimicrobial Susceptibility Testing (EUCAST) recommendations [23]. For all CRE isolates, detection of carbapenemase (*bla*_KPC_, *bla*_NDM_, *bla*_IMP_, *bla*_VIM_, *bla*_OXA-48_) and extended spectrum beta lactamase (ESBL) genes (*bla*_CTX-M-15_, *bla*_TEM_, *bla*_SHV_) was performed by multiplex PCR according to the previously published protocols [24,25]. Carbapenemase-specific PCR products were sequenced with Sanger sequencing technology and obtained sequences were compared using BLAST online available software (https://blast.ncbi.nlm.nih.gov/Blast.cgi?PROGRAM=blastn&BLAST_SPEC=GeoBlast&PAGE_TYPE=BlastSearch, accessed on 15 July 2022).

Molecular typing was conducted by Enterobacterial Repetitive Intergenic Consensus PCR (ERIC PCR) with ERIC-1R (5′-ATGTAAGCTCCTGGGGATTCAC-3′) and ERIC-2 (5′-AAGTAAGTGACTGGGGTGAGCG-3′) primers, as previously described [26]. All available isolates were included in the analysis (37 out of 45 OXA-48 and 30 out of 42 KPC isolates). The amplified products were separated in 2% agarose gel by electrophoresis. The gel was visualized under a UV trans-illuminator for photo-documentation. Phylogenetic analysis of genetic profiles obtained by ERIC-PCR was performed using GelJ software version 2.0 [27]. Clusters were determined based on Pearson correlation coefficient pairwise pattern matching and unweighted pair group method with arithmetic averages (UPGMA). Isolates with above 90% similarity were considered to be clonally related [28].

For statistical analysis, chi square test and univariate logistic regression were utilized to determine the association of different factors with CRE carriage. Mann-Whitney U test was applied for continuous non-parametric variables. All statistical analysis were calculated using SPSS version 21.0 (SPSS Inc. Chicago, IL, USA) software. A two-tailed value of *p* < 0.05 was considered to be statistically significant.

## Figures and Tables

**Table 1:** Characteristics of patients and statistical analysis of variables potentially associated with colonization with carbapenem-resistant Enterobacterales (CRE).

Covariate	N = 350	%	Colonized (N = 88)		Non-Colonized (N = 262)		OR (CI, 95%)	*p* Value
			N	%	N	%		
Male sex	183	52	46	52.2	137	52.2	1.00 (0.617–1.623)	1.001
Premature (≤32 w/g)	175	50	53	60.2	122	46.5	1.72 (0.917–3.241)	0.09
One-minute Apgar score at birth (≤7)	258	73.7	75	85.2	183	69.8	2.967 (1.496–5.887)	0.002
Birth weight ≤ 1000 g	52	14.8	22	25	118	45.4	2.25 (1.289–3.941)	0.004
Small for gestational age	77	22	20	22.7	47	17.9	1.35 (0.75–2.43)	0.348
Mechanical ventilation	139	39.7	40	45.5	99	37.8	1.36 (0,83–2.22)	0.203
Umbilical venous catheter	208	59.4	68	77.2	140	53.3	2.96 (1.70–5.16)	<0.001
Total parenteral nutrition	86	24.6	27	30.6	59	22.5	1.52 (0.89–2.60)	0.152
Retinopathy of prematurity	121	34.6	32	36.4	89	34	1.11 (0.671–1.84)	0.699
Bronchopulmonary dysplasia	18	5.1	5	5.6	13	5	1.15 (0.34–3.32)	0.780
Persistent ductus arteriosus	65	18.6	19	21.6	46	17.5	1.28 (0.70–2.32)	0.432
Necrotizing enterocolitis	16	4.6	10	11.3	6	2,3	5.47 (1.93–15.52)	0.001
Respiratory distress syndrome	282	80.6	77	87.5	205	78.2	1.94 (0.97–3.90)	0.060
Intraventricular hemorrhage	94	26.8	35	39.8	59	22.5	2.27 (1.35–3.80)	0.002
Clinical signs of sepsis (without positive blood culture)	78	22.2	25	28.4	53	20.2	2.53 (1.44–4.46)	0.11
Laboratory confirmed sepsis (positive blood culture)	53	15.1	27	30.6	26	29.3	3.07 (1.72–5.50)	< 0.001
Stay at neonatal intensive care unit	198	56.5	66	75	132	50.3	2.95 (1.72–5.07)	<0.001
Days at neonatal intensive care unit	16.98 ± 19.21		17.32 ± 17.11		15.86 ± 17.12		N.A.	0.403
Days of hospitalization	54.2 ± 34.9		66.28 ± 36.23		50.14 ± 33.63		N.A.	<0.001

OR—Odds ratio; CI—confidence interval; w/g—week of gestation; N.A.—not applicable; *p* values were calculated using chi square test or Fisher test when appropriate; for non-parametric variables Mann-Whitney U test was applied.

**Table 2:** Antibiotic therapy in regard to colonization with carbapenem-resistant Enterobacterales (CRE).

Covariate	N = 350	%	Colonized (N = 88)		Non-Colonized (N = 262)		OR (CI, 95%)	*p* Value
			N	%	N	%		
Initial therapy ≥7 days	109	31.1	25	28.4	84	32.1	0.84 (0.49–1.43)	0.590
Carbapenems	173	49.4	63	71.6	110	42	3.48 (2.06–5.88)	<0.001
Ampicillin/sulbactam + colistin	7	2	1	1.1	6	2.2	0.49 (0.58–4.13)	0.685
Meropenem + ampicillin/sulbactam	89	25.4	32	36.4	57	21.7	2.05 (1.20–3.47)	0.010
Meropenem + vancomycin	43	12.3	17	19.3	26	9.9	2.17 (1.11–4.23)	0.025
Meropenem + colistin	35	10	16	18.1	19	7.2	2.84 (1.39–5.81)	0.006
Meropenem + ceftazidime	19	6.3	12	13.6	7	2.7	5.72 (2.18–15.12)	<0.001

OR—Odds ratio; CI—confidence interval; *p* values were calculated chi square test.

**Table 3:** Distribution of carbapenemase genes, antimicrobial susceptibility and patterns of antimicrobial resistance.

Bacterial Species	*Klebsiella pneumoniae*				*Escherichia coli*
Type of Carbapenemase	*bla*_KPC_ (N = 42)		*bla*_OXA-48_ (N = 45)		*bla*_NDM_ (N = 1)
Number (N) and % of the Isolates	N	%	N	%	N
Antimicrobial resistance					
Piperacillin-tazobactam	42	100	45	100	1
3rd, 4th generation cephalosporins	42	100	45	100	1
Fluoroquinolones	42	100	45	100	0
Gentamicin (G)	5	11.9	45	100	1
Amikacin (AK)	38	90.5	14	31.1	1
Chloramphenicol (CHL)	22	52.4	4	8.9	0
Trimethoprim-sulfamethoxazole (SXT)	22	52.4	4	8.9	0
Colistin	0	0	0	0	0
Prevalent patterns of resistance					
G-R AK-S CHL-S SXT-S	3	7.1	27	60	0
G-R AK-R CHL-S SXT-S	0	0	14	31.1	1
G-S AK-R CHL-R SXT-R	18	42.9	0	0	0
G-S AK-R CHL-S SXT-S	17	40.5	0	0	0

## Data Availability

The data presented in this study are available on request from the corresponding author.
